# Associations between the Timing and Nutritional Characteristics of Bedtime Meals and Sleep Quality for Nurses after a Rotating Night Shift: A Cross-Sectional Analysis

**DOI:** 10.3390/ijerph20021489

**Published:** 2023-01-13

**Authors:** Jung Hoon Park, Hyuntae Park, Seongryu Bae, Jiyeon Kang

**Affiliations:** 1 Neurological Intensive Care Unit, Dong-A University Medical Center, Busan 49201, Republic of Korea; 2 Department of Health Sciences, Graduate School, Dong-A University, Busan 49315, Republic of Korea; 3 College of Nursing, Dong-A University, Busan 49201, Republic of Korea

**Keywords:** sleep disorders, circadian rhythm, shift work schedule, diet, food and nutrition, nurses

## Abstract

The purpose of this study was to investigate the associations of the timing and nutritional characteristics of bedtime meals with sleep quality in nurses after rotating night shifts. In total, 128 nurses from a university hospital in South Korea participated in this cross-sectional study. Data were collected on the first night of two or three consecutive routine night shifts. Participants recorded all food eaten before going to bed after work. An accelerometer was used to objectively measure sleep quality, and subjective sleep quality was assessed by self-report using the Korean version of the Verran and Snyder-Halpern Sleep Scale. The associations of timing and nutritional characteristics of bedtime meals with sleep quality after night shifts were analyzed using multivariate linear regression. A short time interval between meals and sleep was associated with longer objectively measured total sleep time (β = −0.37, *p* = 0.002), and the proportion of protein in meals was associated with better objectively measured sleep efficiency (β = 0.31, *p* = 0.007). The shorter the time interval, the better the subjective sleep quality (β = −0.23, *p* = 0.048), and high-calorie meals were positively associated with subjective sleep quality (β = 0.23, *p* = 0.043). Based on our findings, we encourage nurses to have protein-rich meals after night shifts and reduce the delay between meals and sleep. Although high-calorie meals were shown to have a positive effect on subjective sleep quality, it is necessary to confirm this effect through additional research.

## 1. Introduction

Although sleep is essential to maintaining human function, most shift workers experience poor sleep quality, including sleep disorders such as insufficient total sleep time, lack of deep sleep, and insomnia [1]. Particularly, the prevalence of poor sleep quality in shift-working nurses has been reported to be over 70% [2]. Most Korean nurses are shift workers, and their daily sleep time is generally 354–414 min [3], which is approximately 60−120 min shorter than the average in the general adult population in South Korea [4].

Sleep disturbances can negatively impact shift workers’ health outcomes and work parameters. Based on results from a relevant meta-analysis, Kecklund et al. [5] reported that shift work was associated with several negative health outcomes, including occupational accidents/injuries, type 2 diabetes, coronary heart disease, stroke, and cancer. A lack of sleep for shift-working nurses can also threaten patient and nurse safety [2,6,7], and increase job stress and turnover intention [8]. Specifically, rotating night shifts are known to have more negative effects on health and safety than permanent night shifts [9].

Several factors can affect shift-working nurses’ sleep. Structural equation models for nurses’ sleep quality have identified circadian rhythms, shift work experience, menstrual distress, mood, health status, and sleep hygiene including sleep schedule, arousal-related behaviors, eating/drinking habits prior to sleep, and sleep environment as influencing factors [10,11]. Further, researchers’ interest in dietary intake in relation to sleep hygiene practices has been increasing. Sleep is a major regulator of appetite, blood sugar, and energy metabolism; thus, the relationship between dietary intake and sleep is important [12]. Irregular meals, confectionary foods, and fat intake have been linked to sleep disorders [13,14]. Conversely, high-carbohydrate or Mediterranean diets have been shown to contribute to better sleep [13,15,16].

Breakfast and late-night dinners, both of which can be eaten after night shifts, are important meals for chrono-nutrition, meaning the effect of eating time on health outcomes [12]. Night shift workers tend to eat less healthy meals than workers on morning or afternoon shifts [17]. Bedtime meals after night shifts, high daily caloric intake, and a high proportion of calories in bedtime meals can cause deviations in shift workers’ circadian rhythms [18]. It is therefore important to know what types of foods nurses should eat after night shifts and when they should eat. Recently, Heath et al. [17] reported that high carbohydrate intake and low protein intake were factors that reduced sleep efficiency in shift-working nurses; however, they did not provide specific information on the relationship between bedtime meals and sleep after a night shift.

As described above, the importance of bedtime meals after night shifts has been highly appreciated, but empirical studies on the specific relationship between the two are limited. Therefore, the purpose of this study was to investigate the effects of bedtime meals on nurses’ sleep quality after rotating night shifts. The specific research questions were: (1) does the timing of a meal affect sleep quality after a night shift, and (2) does the calorie and nutritional composition of bedtime meals affect the quality of sleep?

## 2. Materials and Methods

### 2.1. Design

This was an observational study to investigate the effects of bedtime meals on nurses’ sleep quality after a night shift.

### 2.2. Setting and Participants

Nurses working at a university hospital in B city, South Korea participated in the study. This tertiary hospital had approximately 1000 beds and employed about 1200 nurses. Most nurses in the hospital rotated through two or three consecutive night shifts every 10 to 12 days. They were compensated with one day off after the end of consecutive night shifts. The night shift for all nursing departments in the hospital started at 10:00 PM and ended at 7:00 AM the next day. Only nurses scheduled for the first night of consecutive night shifts participated in this study. We excluded nurses who did not perform shift work, were diagnosed with insomnia or a mental health disorder, or drank alcohol or took sleeping pills at the time of the study.

We calculated the sample size using the G power 3.1.9.2 program. For a medium effect size of f = 0.15, significance level of α = 0.05, power of (1 − β) = 0.80, and number of independent variables set to 10, the minimum sample size required for multivariate linear regression analysis was 118. We collected data on bedtime meals and sleep from 128 nurses. For eight nurses, sleep data measured by the accelerometer were not recorded; therefore, we used subjective sleep assessments for 128 nurses and sleep time data for 120 nurses in the final analysis.

### 2.3. Bedtime Meals

Participants self-reported all the food they consumed before going to bed after their first night shift. This included the type of food, brand name, amount, and time of eating. A research assistant calculated the total calories (kcal) and the proportions (%) of protein, carbohydrates, and fat by entering nurses’ food intake records into the CAN-Pro 5.0 program developed by the Korean Nutrition Society.

### 2.4. Sleep Time

We measured participants’ total sleep time (TST), wake after sleep onset (WASO), and sleep efficiency (SE) using a GT3X accelerometer (Actigraph Co., Pensacola, FL, USA). The GT3X is composed of three diaphragms that detect three axial movements of the limbs and calculate acceleration changes in one-minute increments to distinguish between sleep and wakefulness. The device can be worn on the wrist, and is small and lightweight, which minimizes discomfort to the user. In a previous study, the agreement between a GT3X accelerometer and polysomnography was 93% [19], and the validity of the GT3X has been reported in terms of TST, WASO, and SE in both the general healthy population and patient groups [20,21]. The participants were divided into three groups according to their sleep duration: short, <330 min; optimal, 330–480 min; and long, >480 min [22]. The estimated risk of sleep disturbance was defined as poor sleep for short and long sleep duration and better sleep for optimal duration.

### 2.5. Subjective Sleep Assessment

We assessed nurses’ subjective sleep quality with the Korean version of the Verran and Snyder-Halpern (VSH) Sleep Scale [23,24]. The VSH Sleep Scale comprises eight items that assess sleep quality: sleep latency, mid-sleep awakenings, soundness of sleep, movement during sleep, how to wake up, rest upon awakening, subjective quality of sleep, and total sleep period. This self-report tool evaluates each item on a visual analogue scale ranging from 0−10. The total score range is 0−80, and higher scores indicate better sleep quality. At the time of scale development, Cronbach’s α was 0.82 [24]. In the present study, Cronbach’s α = 0.75.

### 2.6. Procedure

We collected data from 10 August to 22 October 2018. After IRB approval, we recruited nurses through a bulletin board and intranet notices. When a nurse showed willingness to participate, a research assistant assessed in advance whether the nurse met the selection criteria. We distributed the accelerometers to the selected participants and explained how to use the device and research procedures for approximately 10 min. Participants wore an accelerometer on their wrists from the beginning of the first night shift. They recorded all snacks or meals they consumed between finishing the night shift and going to sleep. After waking up, they removed the accelerator and assessed subjective sleep quality using the VSH Sleep Scale. A research assistant downloaded and analyzed the sleep time data recorded by the accelerators.

### 2.7. Ethical Considerations

The Institutional Review Board of Dong-A University approved the study protocol (approval number: 2-1040709-AB-N-01-201805-HR-007-02). All participants voluntarily decided to participate and provided written informed consent.

### 2.8. Statistical Analysis

We analyzed the data using IBM SPSS version 24.0 (IBM Corp.; Armonk, NY) and R Statistical Software (R Studio version 1.3.1093; R Foundation for Statistical Computing, Vienna, Austria). Participants’ characteristics, food intake, sleep time, and subjective sleep assessment were presented as means and standard deviations (SD). Data were tested for normality (Kolmogorov–Smirnov test), and the differences in sleep according to participant characteristics were analyzed by t-test and one-way analysis of variance. We analyzed correlations between bedtime meal timing, nutrient proportions, sleep time, and subjective sleep by calculating Pearson correlation coefficients. Multivariate regression analyses were performed to identify factors associated with sleep quality after a night shift. Independent variables were the participant characteristics that were significant in the univariate analyses. The main variables, bedtime meal characteristics such as time interval between having a meal and sleep quality, proportions of protein and fat, and total calories were also used as independent variables in the regression models, regardless of univariate results. However, the proportion of carbohydrates was excluded because it was highly correlated with proportions of both protein and fat. In all regression models, values of the variance inflation factor were 1.03–1.18, which were less than 10; therefore, there was no problem of multicollinearity.

The generalized additive model (GAM) was compared with the generalized linear model based on the Akaike information criterion. All Akaike information criterion results showed that the GAM had a better model fit in our sample [25]. GAM is an extensive mathematical model of the generalized linear model. This model is a nonparametric regression technique that is not restricted by linear associations [26]. The GAM replaces the linear predictor with an additive one that can model continuous data as a nonlinear smoothing function and estimate it as part of the fitting. We specified the negative binomial distribution in the GAMs to examine the dose–response relationships between total energy intake, time interval between the meal and sleep, and the partial effect of estimated risk of sleep disturbance. We initially conducted univariate GAM testing and added age, gender, marital status, nutrition status, and body mass index as covariates. All data were interpreted as the mean ± SD and the significance level was set at 0.05.

## 3. Results

### 3.1. Characteristics of Participants and Their Bedtime Meals and Sleep after Night Shifts

A total of 128 rotating night-shift nurses participated in this study, and their characteristics are shown in Table 1. Most participants were women (93.8%), and the mean age of the participants was 26.90 ± 5.69 years. Their mean clinical experience was 55.40 ± 62.72 months, and the average number of night shifts per month was 6.50 ± 1.24.

On average, participants consumed 428.20 ± 262.23 kcals before going to sleep after a night shift. The mean proportions of protein, fat, and carbohydrates of the meals were 12.82% ± 6.56, 27.53% ± 15.31, and 59.54% ± 18.53, respectively. They went to bed approximately 1.43 ± 1.18 h after eating. Their TST was 334.97 ± 102.44 min, WASO was 17.64 ± 19.07 min, and SE was 95.35% ± 4.69. Regarding subjective sleep assessment, the mean VSH Sleep Scale score was 38.96 ± 10.71 (Table 2).

### 3.2. Differences in Sleep Quality According to Participant Characteristics

TST showed gender differences (t = 2.42, *p* = 0.017), as the TST of men (432.50 ± 129.8) was significantly longer than that of women (332.41 ± 96.97). WASO differed according to the number of night shifts per month (t = 2.47, *p* = 0.015). The WASO of participants who worked less than seven nights per month (19.94 ± 19.86) was significantly longer than those who worked more than seven nights (10.10 ± 12.31). There were no significant differences in SE according to participant characteristics.

There were significant differences in the subjective assessment of sleep quality according to age (t = 2.14, *p* = 0.035) and shift work experience (t = 2.10, *p* = 0.037). The mean VSH sleep score of participants younger than 27 years old (40.87 ± 10.00) was significantly higher than those age 27 and older (36.66 ± 10.94). The mean VSH sleep score of participants with less than three years of clinical experience (41.63 ± 10.04) was significantly higher than those with three years or more of clinical experience (37.71 ± 10.54).

### 3.3. Correlations between Bedtime Meals and Sleep Measures

There was a positive correlation between the proportions of protein and fat in meals before bedtime (r = 0.32, *p* < 0.01). Additionally, there were negative correlations between the proportions of carbohydrates and fat (r = −0.94, *p* < 0.001) and of carbohydrates and protein (r = −0.62, *p* < 0.001).

The higher the proportion of protein, the higher the SE (r = 0.28, *p* < 0.05). The longer the time interval between meals and sleep, the shorter the TST (r = −0.36, *p* < 0.01). The longer the WASO, the lower the SE (r = −0.70, *p* < 0.001). There were no significant correlations between subjective sleep assessment and meal characteristics or sleep time (Table 3).

### 3.4. Factors Associated with Sleep after a Night Shift

We conducted multivariate regression analyses for TST, WASO, SE, and the subjective sleep assessment, and the results are shown in Table 4.

The only significant factor associated with TST was the time interval between bedtime meals and sleep; as the time interval decreased, TST increased (β = −0.37, *p* = 0.002). No factors were significantly associated with WASO. The factor that was significantly associated with SE was protein proportion; the higher the proportion of protein in the bedtime meals, the higher the SE (β = 0.31, *p* = 0.007).

Both the time interval between bedtime meals and sleep and total calories per meal were significantly associated with the subjective assessment of sleep quality. The shorter the time interval, the better the subjective sleep assessment (β = −0.23, *p* = 0.048). Additionally, high-calorie meals were positively associated with subjective sleep assessment such as VSH sleep score (β = 0.23, *p* = 0.043).

**Figure 1:**
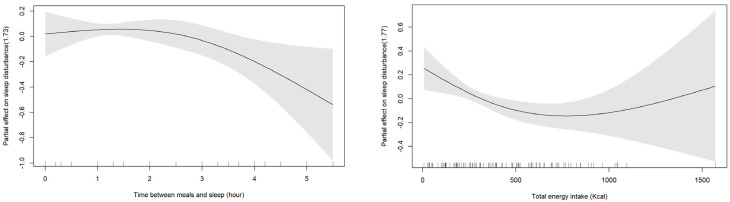
Estimated dose–response relationships of total energy intake and time interval between meal and sleep with estimated risk of sleep disturbance in the total sample. The model was adjusted for age, gender, marital status, and body mass index.

Figure 1 shows the predicted relationship between duration of total energy intake and time interval between meal and sleep, and the partial effect of estimated risk of sleep disturbance. After adjusting for age, gender, marital status, nutrition status, and body mass index, we found a curvilinear relationship between the two parameters and sleep disturbance risk. The model revealed a negative exponential relationship between time between having a meal and sleep and the risk of sleep disturbance in the full-adjusted GAM model. The meal to sleep time interval showed that around 1.74 h had the highest partial effects on the risk of developing sleep disturbance (0.51; standard error: 0.03) (degrees of freedom = 1.73). In addition, a U-shaped relationship between total energy intake and the risk of developing sleep disturbance was noted (degrees of freedom = 1.77), with the lowest partial effects on the risk of developing sleep disturbance (0.48; standard error: 0.04) when total energy intake was 523 kcal.

## 4. Discussion

We conducted this study to investigate the association between bedtime meals and sleep quality in nurses after rotating night shifts. We found total energy intake and short time interval between meals and sleep was linearly and non-linearly associated with longer objectively measured total sleep time, and the proportion of protein in meals was associated with better objectively measured sleep efficiency. In addition, shorter time intervals between meals and sleep and high-calorie meals were associated with better subjective sleep quality.

Participating nurses’ TST after a night shift was approximately 334 min, which is similar to the TST of night shift workers reported in previous studies [3,27]. However, this was shorter by two hours than the 471 min average TST for healthy Korean adults [4]. The other two accelerometer measurements met the criteria for normal sleep: WASO < 30 min and SE > 85% [28]. These results indicated that after a night shift, sleep deviates from the body’s normal circadian rhythms [18]; thus, TST is shortened, but WASO decreases due to fatigue caused by working the night shift [29].

There was no significant correlation between subjective assessment of sleep quality and sleep time as measured by the accelerometer. Differences between self-assessments and accelerometer evaluations have also been reported in other recent studies [30,31,32]. Zhang et al. [33] noted there may be differences between objective and subjective measures for sleep disorders, and clinical studies need to consider both indicators. Therefore, they should be considered as different sleep quality parameters, and it seems reasonable to consider subjective sleep assessment as individual satisfaction with one’s sleep.

The core finding of this study was that the proportion of protein in bedtime meals was positively associated with SE. Sleep is a major factor that affects the body’s internal clock, and these effects can be mediated by food intake [12]. The relationship between protein intake and sleep quality has also been reported in previous studies. High school girls who consumed protein-rich snacks were shown to have better sleep quality [3]. Additionally, low protein intake in shift-working nurses has been shown to be associated with low sleep quality [17]. Our study differs from these two studies reporting the effects of daily protein intake on sleep, in that the bedtime protein intake increased participants’ SE after a night shift.

Among protein components, tryptophan is an amino acid that is well known to be a sleep-promoting factor, and is abundant in milk, eggs, soybeans, and chicken [34,35]. In the brain, tryptophan is converted to serotonin, a precursor of the sleep-inducing hormone melatonin, to promote sleep [36]. Most protein-rich foods also contain a lot of vitamin B, and vitamin B is also considered to be a sleep-promoting nutrient [35,37]. Additionally, protein can improve SE by inhibiting gastric acid reflux during sleep [38]. Nevertheless, nurses tend to consume less protein during the night shift, compared to during the morning shift [17]. Nurses are generally aware that they need a healthy diet to effectively deal with the negative effects of shift work; however, a recent study showed that few nurses tried to eat healthily [1]. Unfortunately, specific information and guidance on the types of foods that are best for nurses to eat when working the night shift is limited. However, based on the results of the present study, having a protein-rich meal may help to improve SE in nurses after working a night shift

Under normal circadian rhythms, the longer the time interval between meals and sleep, the higher the quality of sleep, since eating before sleep has a negative effect on gastrointestinal movement and metabolic processes [34,39]. However, we observed that a short time interval between meals and sleep increased TST in the present study. This can be interpreted as a variation from an ordinary situation because nurses’ meals and sleep after night shifts are against normal circadian rhythms. Most previous studies only reported the relationship between daily diet or nutritional status and sleep, not eating time [12,35]. The sleep–wake cycle is jointly determined by two processes: Process C represents the sleep pressure generated by circadian rhythms, and Process S is defined as a homeostatic process that interacts with Process C [40]. Shift workers may experience circadian misalignment, and there has been an increased understanding of how milder shifts in eating and sleeping patterns can have adverse health consequences. Sleep displacement and altered meal timing due to shift work disturb hormonal balance [41] and glucose metabolism [42], and a long-term consequence of altered glucose metabolism shift work is associated with an increased risk of type 2 diabetes [43]. Furthermore, meal timing in association with chronotype such as morning or evening type were shown to modulate the risk associated with late eating [44]. In particular, little research has been conducted on the effects of eating time on morning sleep after a night shift. Therefore, follow-up studies on the proper timing of shift workers’ bedtime meals are needed. A systematic review found that among shift workers, morning types had poorer sleep during night shifts, while evening types had impaired sleep during morning shifts [45]. Several studies have shown that chronotype affects sleep duration in shift workers depending on the morning or night shift [45,46,47]. Shorter sleep duration is related to higher total energy and fat intake, and with lower protein intake [48]. It is possible that the differences in habitual dietary intake between day workers and rotating shift workers may be caused by differences in chronotype [49]. Therefore, it is important to measure chronotype over the 24 h day using actigraphy data because meal times and sleep quality in different shift types are affected by chronotype.

In this study, the higher the total calories of bedtime meals, the better the sleep quality (based on nurses’ subjective sleep assessment). This differed from the findings of previous studies that indicated food intake before sleep negatively impacts sleep quality. For example, Crispim et al. [50] observed that the higher the total calories in women’s bedtime meals, the longer it took them to fall asleep, the greater the number of WASOs, and the lower the SE. Lowden et al. [51] reported that consuming more than 20% of one’s daily calories before bedtime had a negative effect on sleep, and a study of hospital shift workers found a tendency to deviate from normal circadian rhythms as the total calories in bedtime meals increased [18]. Notably, in our study, total calories consumed before bedtime was positively associated with subjective sleep quality, and the possible reason could be psychological satisfaction from food intake. Night shifts cause hunger and anxiety, and food intake can lower anxiety levels [27]. Although our results showed that the higher the total calories of bedtime meals, the better the sleep quality, the average total calorie intake in this study was 428 kcals per meal, which is not high enough to disturb sleep. Generally, the recommended daily calorie intake for women and men in their 20s in South Korea is 2000 and 2600 calories a day, respectively. In other words, the recommended calorie intake is 600–800 calories for each of three meals—breakfast, lunch, and dinner [52]. However, food intake shortly before bedtime is not recommended, because it is associated with metabolic problems such as obesity and diabetes [53]. Therefore, further research is needed on the total calorie intake, anxiety, and sleep quality of shift-working nurses.

The main strength of this study is that this is the first study to use the GAM model to evaluate the linear and nonlinear associations of energy intake and time intervals between meals and sleep and the risk of developing sleep disturbance in nurses. Moreover, the significance of this study is that it suggests a rudimentary direction for the types of foods to eat and meal timing for nurses after a night shift. The present findings can stimulate the development of interventions and guidelines related to eating and sleep for rotating night shift nurses. However, this study also has some limitations that should be noted. First, this study was conducted in one tertiary hospital in South Korea; thus, there is a limit to the generalizability of the results. Second, we observed only the first night shift; therefore, we were unable to provide information about eating and sleeping over consecutive night shifts. Third, we did not control for other variables that could affect sleep quality, such as nurses’ morningness–eveningness, activities, underlying diseases, and workload. Further studies controlling for these variables are warranted. Furthermore, it would also be meaningful to compare whether the nutritional characteristics of bedtime meals and sleep quality after a night shift differ from daily routine. Future studies using actigraphy are needed to compare weekend or free days with night shifts for the same population of nurses. Fourth, an additional limitation was a one-time food survey after the night shift and evaluation of sleep by one-night measurement. It is difficult to determine reliable sleep patterns from a one-time measurement, because sleep is a complex and dynamic biological process. Previous studies using actigraphy reported that measures of sleep duration and efficiency may require five nights or more to obtain adequate estimates of stable individual differences [54]. A recent review into the objective assessment of sleep patterns among night-shift workers found that actigraph devices and Actiwatch were the most common wearable devices used to measure sleep patterns. Sleep duration assessment using the wearable devices in night-shift workers was diverse, ranging from one day to four weeks or longer, and more than half of the studies collected data for over two weeks [55]. The review also suggested that the combined use of subjective assessment with self-report questionnaires in addition to objective sleep measurement would be desirable to assess shift workers’ sleep [55]. Although we assessed the quantity and quality of sleep using both objective and subjective assessments, we still believe that the one-night assessment was insufficient to accurately determine sleep patterns. In the future, it will be necessary to evaluate not only sleep but also food intake over multiple days. Additionally, it is possible that wearing the accelerometer itself could have affected participants’ sleep quality. Lastly, because this was a short-term cross-sectional study, we are unable to determine the causal relationship between food intake and sleep. In future studies, some of the limitations of the present study can be addressed through implementing a rigorous research design that extends the observation period and sample size.

## 5. Conclusions

The present study provides evidence that protein intake prior to bedtime could have a positive impact on sleep efficiency; however, protein intake had no relationship to sleep duration. Moreover, a short time interval between meals and sleep, and total energy intake before sleep, were negatively and non-linearly associated total sleep time.

These findings suggest that earlier timing of eating or lower energy and optimal protein intake in relation to bedtime increases the likelihood of optimal sleep duration. Future investigations of specific foods in relation to sleep duration and quality could generate nutritional recommendations that benefit sleep health. Although cross-sectional data cannot determine cause-and-effect relationships, professional nursing organizations and institutions can use these findings to establish guidelines or recommendations for meal timing and types of foods eaten after night shifts.

## Figures and Tables

**Table 1:** Characteristics of participants and their bedtime meals and sleep (n = 128).

Variables	Categories	n (%)	Mean ± SD
Gender	Man	8 (6.3)	
	Woman	120 (93.8)	
Age (years)	<27	85 (66.4)	26.90 ± 5.69
	≥27	43 (33.6)	
Body Mass Index (kg/m^2^)	<18.5	20 (15.6)	21.00 ± 3.06
	18.5~23	84 (65.6)	
	≥23	24 (18.8)	
Marital status	Married	24 (18.8)	
	Unmarried	104 (81.3)	
Clinical experience (months)	<36	68 (53.1)	55.40 ± 62.72
	≥36	60 (46.9)	
Number of night shifts per month	<7	74 (69.8)	6.50 ± 1.24
	≥7	32 (30.2)	
Position	Staff nurse	126 (98.4)	
	Charge nurse	2 (1.6)	
Department	Inpatient units	56 (14.8)	
	Intensive care units	61 (47.7)	
	Emergency rooms	9 (7.0)	
Bedtime meal	Protein (%)		12.82 ± 6.56
	Fat (%)		27.53 ± 15.31
	Carbohydrate (%)		59.54 ± 18.53
	Total Calorie (Kcal)		428.20 ± 262.23
Time interval between meal and sleep (hours)			1.43 ± 1.18
Accelerometer measured sleep times	Total sleep time (minutes)		334.97 ± 102.44
	Wake After Sleep Onset (minutes)		17.64 ± 19.07
	Sleep Efficiency (%)		95.35 ± 4.69
Subjective sleep assessment	VSH sleep scale score		38.96 ± 10.71

Values are presented as mean ± SD or n (%).

**Table 2:** Sleep differences according to participant characteristics.

Variables	Categories	Total Sleep Time		WASO		Sleep Efficiency		VSH Sleep Scale Score	
		Mean ± SD	t or F	Mean ± SD	t or F	Mean ± SD	t or F	Mean ± SD	t or F
Gender	Man	432.50 ± 129.8	2.42 *	18.86 ± 23.18	0.17	94.20 ± 6.48	−0.70	40.00 ± 14.04	0.14
	Woman	332.41 ± 96.97		17.57 ± 18.90		95.47 ± 4.58		39.44 ± 10.29	
Age (years)	<27	333.49 ± 95.75	−0.60	18.71 ± 20.36	0.83	95.50 ± 4.82	0.31	40.87 ± 10.00	2.14 *
	≥27	345.38 ± 110.4		15.67 ± 16.44		95.21 ± 4.47		36.66 ± 10.94	
Body Mass Index (kg/m^2^)	<18.5	339.84 ± 80.63	0.09	19.45 ± 24.67	0.14	95.85 ± 3.37	0.16	41.00 ± 12.68	0.25
	18.5~23	334.87 ± 103.8		17.04 ± 17.31		95.38 ± 4.95		39.11 ± 10.01	
	≥23	345.38 ± 109.4		18.19 ± 20.28		95.01 ± 4.89		39.50 ± 10.44	
Marital status	Married	338.04 ± 106.0	0.03	19.04 ± 17.71	0.40	95.24 ± 3.44	−0.18	36.71 ± 10.66	−1.45
	Unmarried	337.47 ± 99.87		17.29 ± 19.46		95.43 ± 4.96		40.14 ± 10.36	
Clinical experience (months)	< 36	334.40 ± 101.2	−0.36	18.81 ± 20.03	0.69	95.29 ± 4.98	−0.25	41.63 ± 10.04	2.10 *
	≥36	341.24 ± 101.0		16.40 ± 18.06		95.50 ± 4.38		37.71 ± 10.54	
Number of night shifts per month	< 7	324.38 ± 93.41	−0.71	19.94 ± 19.86	2.47 *	94.92 ± 4.98	−1.67	39.36 ± 11.28	−0.15
	≥7	340.41 ± 114.3		10.10 ± 12.31		96.67 ± 4.04		39.65 ± 9.28	
Position	Staff nurse	337.74 ± 101.4	0.12	17.46 ± 18.83	−0.81	95.39 ± 4.71	−0.11	39.48 ± 10.55	−0.01
	Charge nurse	329.00 ± 65.05		28.50 ± 38.89		95.76 ± 4.11		39.50 ± 0.71	
Department	Inpatient units	347.14 ± 93.27	0.48	17.94 ± 19.83	0.38	95.45 ± 4.70	0.06	39.75 ± 10.91	0.04
	Intensive care units	332.02 ± 107.5		18.19 ± 18.97		95.27 ± 4.60		39.24 ± 9.81	
	Emergency rooms	318.11 ± 103.7		12.33 ± 15.91		95.82 ± 5.60		39.33 ± 12.90	

WASO, Wake After Sleep Onset; VSH, Verran and Snyder-Halpern. * *p* < 0.05.

**Table 3:** Correlations between bedtime meals and sleep.

	1	2	3	4	5	6	7	8	9
1. Time between meals and sleep	1								
2. Protein ratio	0.19	1							
3. Fat ratio	0.16	0.32 **	1						
4. Carbohydrate ratio	−0.20	−0.62 ***	−0.94 ***	1					
5. Total calories	0.16	0.11	0.10	−0.12	1				
6. Total Sleep Time	−0.36 **	−0.00	0.01	−0.01	−0.07	1			
7. Wake After Sleep Onset	−0.11	−0.14	0.02	0.03	0.04	0.04	1		
8. Sleep efficiency	0.19	0.28 *	−0.10	−0.01	−0.00	−0.12	−0.70 ***	1	
9. VSH sleep scale score	−0.12	−0.01	−0.12	0.10	0.19	0.18	0.14	−0.09	1

*** *p* < 0.001, ** *p* < 0.01, * *p* < 0.05.

**Table 4:** Factors affecting sleep quality after night shift.

Sleep	Factors	B	SE	*β*	t	*p*	VIF
Total Sleep Time R^2^ = 0.151 F = 2.532 *p* = 0.036	(constant)	500.21	110.57		4.52	<0.001	
	Gender	−62.03	51.48	−0.14	−1.20	0.232	1.12
	Time interval between meals and sleep (hour)	−30.73	9.39	−0.37	−3.27	0.002	1.06
	Protein ratio (%)	0.89	1.89	0.05	0.47	0.640	1.10
	Fat ratio (%)	0.24	0.75	0.04	0.32	0.752	1.07
	Total calories (Kcal)	−0.03	0.05	−0.07	−0.56	0.579	1.18
Wake After Sleep Onset R^2^ = 0.035 F = 0.545 *p* = 0.741	(constant)	21.88	13.27		1.65	0.103	
	Monthly number of night shifts	0.01	1.68	0.00	0.01	0.996	1.03
	Time interval between meals and sleep (hour)	−1.62	1.88	−0.10	−0.86	0.391	1.07
	Protein ratio (%)	−0.47	0.39	−0.15	−1.22	0.227	1.10
	Fat ratio (%)	0.08	0.16	0.06	0.50	0.622	1.11
	Total calories (Kcal)	0.01	0.01	0.08	0.68	0.500	1.05
Sleep Efficacy R^2^ = 0.140 F = 3.040 *p* = 0.022	(constant)	93.15	1.56		59.68	<0.001	
	Time interval between meals and sleep (hour)	0.76	0.45	0.19	1.71	0.091	1.06
	Protein ratio (%)	0.26	0.09	0.31	2.77	0.007	1.09
	Fat ratio (%)	−0.06	0.04	−0.17	−1.53	0.130	1.05
	Total calories (Kcal)	−0.00	0.00	−0.11	−0.97	0.337	1.09
Subjective Assessment R^2^ = 0.206 F = 2.981 *p* = 0.012	(constant)	49.97	5.26		9.51	<.001	
	Age (year)	−5.18	3.13	−0.25	−1.65	0.103	1.93
	Clinical experience (month)	−1.40	3.03	−0.07	−0.46	0.646	1.87
	Time interval between meals and sleep (hour)	−1.89	0.94	−0.23	−2.02	0.048	1.11
	Protein ratio (%)	0.07	0.19	0.04	0.36	0.724	1.10
	Fat ratio (%)	−0.13	0.08	−0.19	−1.66	0.102	1.12
	Total calories (Kcal)	0.01	0.01	0.23	2.06	0.043	1.09

VIF, variance inflation factor.

## Data Availability

Qualified researchers can obtain the data from the corresponding author (jykang@dau.ac.kr). The data are not publicly available due to privacy concerns imposed by the IRB.
